# SUMOylation of the m6A reader YTHDF2 by PIAS1 promotes viral RNA decay to restrict EBV replication

**DOI:** 10.1128/mbio.03168-23

**Published:** 2024-01-18

**Authors:** Febri Gunawan Sugiokto, Farjana Saiada, Kun Zhang, Renfeng Li

**Affiliations:** 1 Department of Oral and Craniofacial Molecular Biology, School of Dentistry, Virginia Commonwealth University, Richmond, Virginia, USA; 2 Program in Microbiology and Immunology, University of Pittsburgh, Pittsburgh, Pennsylvania, USA; 3 Department of Microbiology and Molecular Genetics, University of Pittsburgh, Pittsburgh, Pennsylvania, USA; 4 Philips Institute for Oral Health Research, School of Dentistry, Virginia Commonwealth University, Richmond, Virginia, USA; 5 Department of Microbiology and Immunology, School of Medicine, Virginia Commonwealth University, Richmond, Virginia, USA; 6 Massey Cancer Center, Virginia Commonwealth University, Richmond, Virginia, USA; 7 Cancer Virology Program, Hillman Cancer Center, University of Pittsburgh Medical Center, Pittsburgh, Pennsylvania, USA; The University of North Carolina at Chapel Hill, Chapel Hill, North Carolina, USA

**Keywords:** PIAS1, YTHDF2, restriction factor, Epstein-Barr virus, SUMOylation, m6A, RNA modification, lytic replication, RNA decay

## Abstract

YTH N6-methyladenosine RNA-binding protein F2 (YTHDF2) is a member of the YTH protein family that binds to N6-methyladenosine (m6A)-modified RNA, regulating RNA stability and restricting viral replication, including Epstein-Barr virus (EBV). PIAS1 is an E3 small ubiquitin-like modifier (SUMO) ligase known as an EBV restriction factor, but its role in YTHDF2 SUMOylation remains unclear. In this study, we investigated the functional regulation of YTHDF2 by PIAS1. We found that PIAS1 promotes the SUMOylation of YTHDF2 at three specific lysine residues (K281, K571, and K572). Importantly, PIAS1 synergizes with wild-type YTHDF2, but not a SUMOylation-deficient mutant, to limit EBV lytic replication. Mechanistically, YTHDF2 lacking SUMOylation exhibits reduced binding to EBV transcripts, leading to increased viral mRNA stability. Furthermore, PIAS1 mediates SUMOylation of YTHDF2’s paralogs, YTHDF1 and YTHDF3, to restrict EBV replication. These results collectively uncover a unique mechanism whereby YTHDF family proteins control EBV replication through PIAS1-mediated SUMOylation, highlighting the significance of SUMOylation in regulating viral mRNA stability and EBV replication.

**IMPORTANCE:**

m6A RNA modification pathway plays important roles in diverse cellular processes and viral life cycle. Here, we investigated the relationship between PIAS1 and the m6A reader protein YTHDF2, which is involved in regulating RNA stability by binding to m6A-modified RNA. We found that both the N-terminal and C-terminal regions of YTHDF2 interact with PIAS1. We showed that PIAS1 promotes the SUMOylation of YTHDF2 at three specific lysine residues. We also demonstrated that PIAS1 enhances the anti-EBV activity of YTHDF2. We further revealed that PIAS1 mediates the SUMOylation of other YTHDF family members, namely, YTHDF1 and YTHDF3, to limit EBV replication. These findings together illuminate an important regulatory mechanism of YTHDF proteins in controlling viral RNA decay and EBV replication through PIAS1-mediated SUMOylation.

## INTRODUCTION

Host restriction factors play a crucial role in defending against viral infection, replication, and egress by targeting viral proteins, viral DNA, and/or RNA (1). One restriction factor YTH N6-methyladenosine RNA-binding protein F2 (YTHDF2) is an RNA-binding protein that specifically recognizes and binds to N6-methyladenosine (m6A)-modified RNA (2, 3). Studies from our lab and other groups have highlighted the key role of YTHDF2 in limiting the reactivation of Epstein-Barr virus (EBV) and Kaposi’s sarcoma-associated herpesvirus (KSHV) through targeting and destabilizing viral and cellular RNAs (4–7). YTHDF2 has also been implicated in destabilizing hepatitis B virus mRNA in coordination with ISG20 (8). Our recent study has shown that caspase-mediated cleavage of YTHDF2 antagonizes its anti-viral activity during EBV reactivation process (7).

In addition to cleavage, YTHDF2 can also be regulated through phosphorylation, ubiquitination, and SUMOylation. Phosphorylation of YTHDF2 at S39 and T381 by the EGFR/SRC/ERK signaling pathway contributes to its stabilization (9). The stability of YTHDF2 is also regulated by the activity of cyclin-dependent kinase 1 (CDK1), where phosphorylated YTHDF2 can be targeted for ubiquitination-dependent degradation by E3 ubiquitin ligase complexes, including Cullin 1, Cullin 4A, damaged DNA-binding protein 1, and S-phase kinase-associated protein 2 (10). These studies highlight the significance of post-translational modifications in modulating the function of YTHDF2 in mRNA regulation, particularly in the context of m6A modification.

SUMOylation is a post-translational modification (PTM) process involving the attachment of small ubiquitin-like modifier (SUMO) proteins (SUMO1/SUMO2/SUMO3) to target proteins. This process relies on a cascade of enzymes, including the E1 activating enzyme complex SAE1/SAE2, the E2 conjugating enzyme UBC9, and specific E3 protein ligases (11). Among the E3 ligases, the protein inhibitor of activated family proteins (PIAS1, PIASx/PIAS2, PIAS3, and PIASy/PIAS4) are known to be involved in SUMOylation (12).

Our previous studies have identified PIAS1 as a restriction factor for EBV (13) and as an E3 ligase to synergize with SAMHD1 to control EBV replication through SUMOylation (14). In this study, we demonstrated that PIAS1 synergizes with YTHDF2 to limit EBV lytic replication. We showed that PIAS1 promotes YTHDF2 SUMOylation at three lysine residues, K281, K571, and K572. The interaction between YTHDF2 and PIAS1, as well as the SUMOylation process mediated by PIAS1, is extended to its paralogs YTHDF1 and YTHDF3, highlighting the significance of PIAS1-mediated SUMOylation in the regulation of m6A readers and the m6A RNA modification pathway.

## RESULTS

### PIAS1 promotes YTHDF2 SUMOylation

In our previous studies, we have shown that both YTHDF2 and PIAS1 play crucial roles in restricting EBV replication (7, 13, 15). YTHDF2 contributes to the decay of viral and cellular genes, while PIAS1 inhibits viral gene transcription. PIAS1, as an E3 SUMO ligase, is responsible for the SUMOylation of various proteins. Recently, we have discovered that PIAS1 promotes the SUMOylation of SAMHD1 on multiple lysine residues to enhance its anti-viral activity (14).

**Fig 1:**
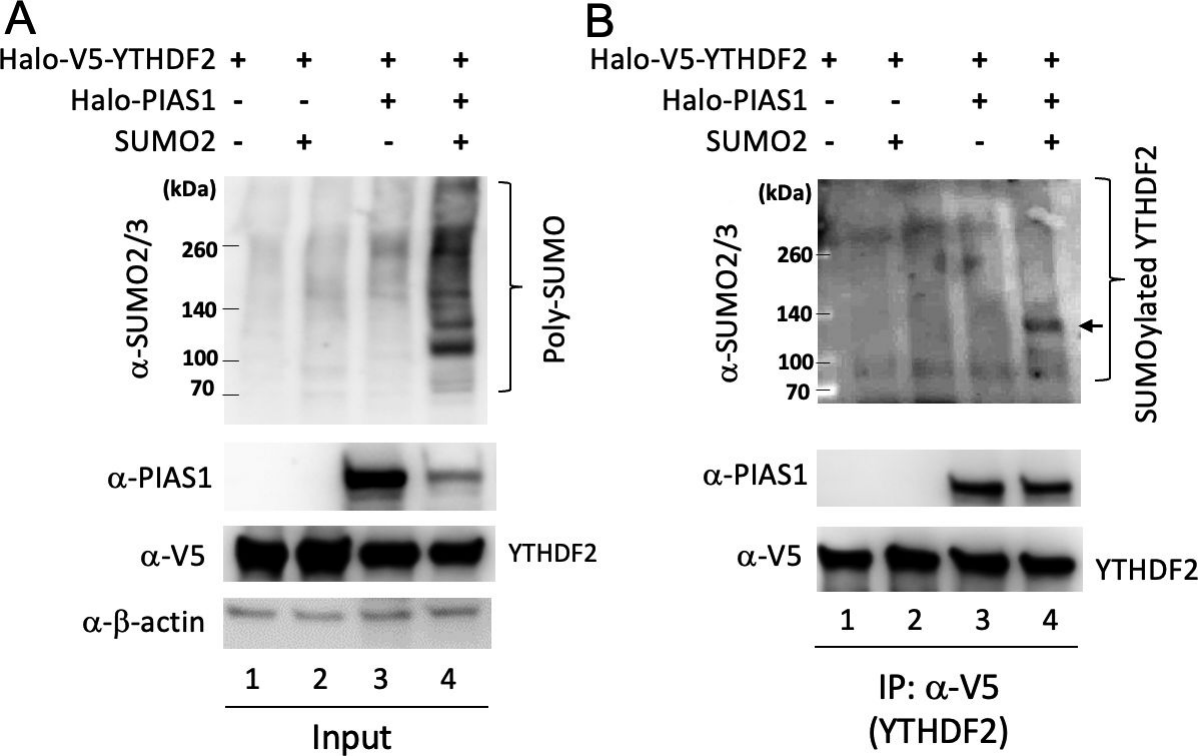
PIAS1 promotes YTHDF2 SUMOylation. HEK293T cells were transfected with Halo-V5-YTHDF2, Halo-PIAS1, and SUMO2 plasmids as indicated. (**A**) WB was performed for the whole-cell lysate (input) using anti-SUMO2/3, anti-PIAS1, anti-V5, and anti-β-actin antibodies. Bracket denotes SUMOylated proteins. (**B**) YTHDF2 was IPed with anti-V5 magnetic beads, and WB was performed using antibodies as indicated. Bracket denotes SUMOylated YTHDF2. IP, immunoprecipitation.

A recent study identified YTHDF2 as a SUMOylated protein (16). However, the E3 ligase responsible for YTHDF2 SUMOylation is not known. We hypothesize that PIAS1 functions as an E3 ligase facilitating YTHDF2 SUMOylation. To test our hypothesis, we transfected HEK293T cells with plasmids expressing YTHDF2, PIAS1 and SUMO2. We found that transfection of SUMO2 or PIAS1 slightly increases the total SUMOylation level (Fig. 1A; top panel, lanes 2 and 3 vs lane 1), while SUMO2 and PIAS1 co-transfection significantly promotes SUMOylation (Fig. 1A; top panel, lane 4). To determine whether PIAS1 promotes the SUMOylation of YTHDF2, we then immunoprecipitated (IPed) YTHDF2 by anti-V5 antibody-conjugated magnetic beads. Western blotting (WB) analysis with anti-SUMO2/SUMO3 antibody showed that YTHDF2 SUMOylation is increased when SUMO2 or PIAS1, or SUMO2 and PIAS1 are expressed (Fig. 1B; top panel, lanes 2–4 vs lane 1), suggesting YTHDF2 is targeted for SUMOylation by PIAS1.

### PIAS1 interacts with YTHDF2

**Fig 2:**
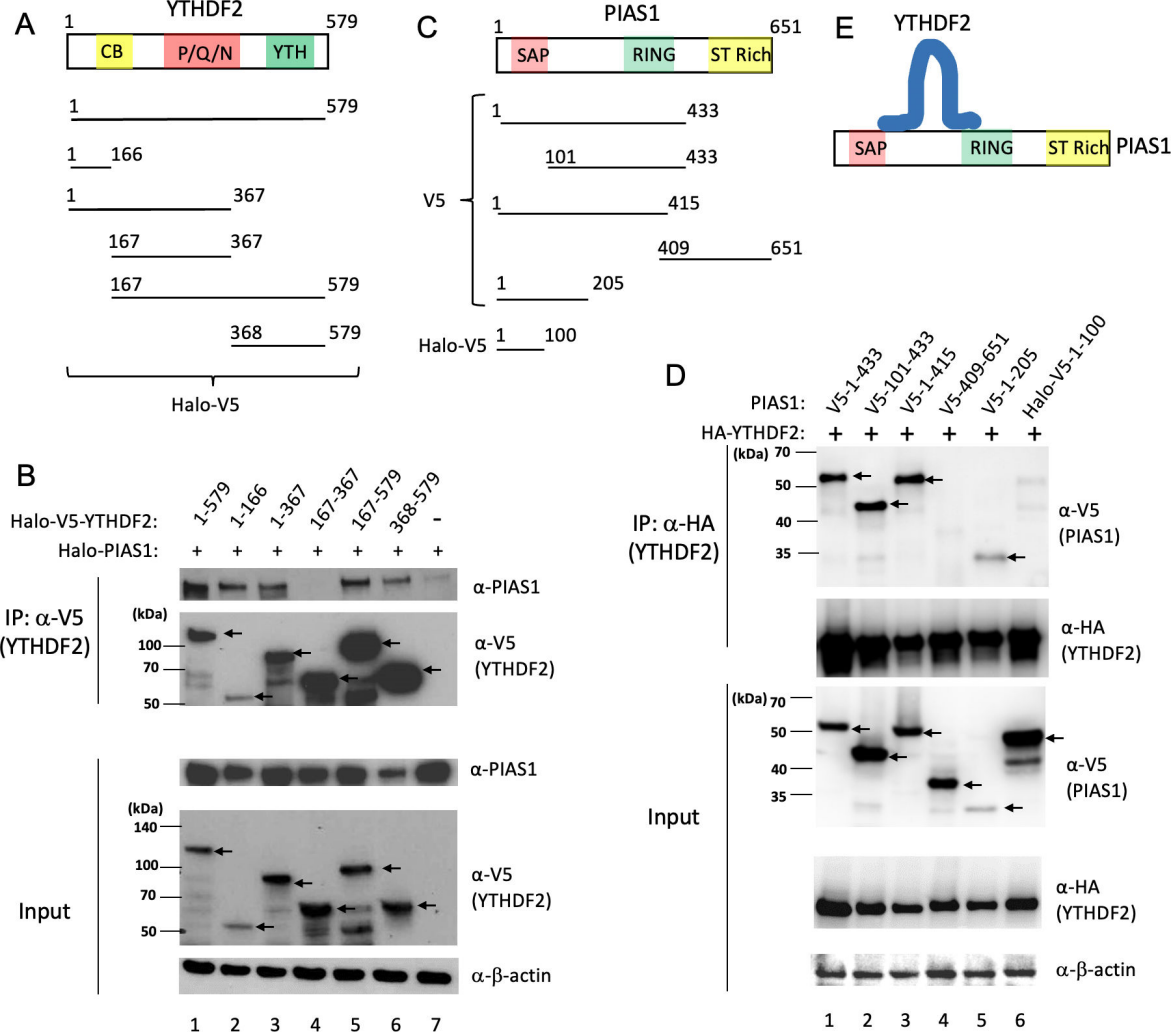
PIAS1 interacts with YTHDF2. (**A**) Illustration of full-length YTHDF2 (1–579) or YTHDF2 truncation mutants. CB denotes CNOT1-binding domain; P/Q/N is a P/Q/N rich aggregation-prone region; YTH denotes m6A RNA-binding domain. (**B**) HEK293T cells were co-transfected with full-length YTHDF2 and truncated YTHDF2 plasmids as indicated. WB analyses showing that PIAS1 is Co-IPed with N-terminal and C-terminal regions of YTHDF2. β-Actin blot was included as loading control. Arrows denote the position of full-length and truncated YTHDF2. (**C**) The schematic representation showing PIAS1 domains, V5-tagged or Halo-V5 tagged PIAS1 truncation mutants. SAP (SAF-A/B, Acinus, and PIAS) is a DNA and protein-binding domain; PINIT denotes the nuclear localization motif; RING indicate RING finger E3 ligase domain for protein SUMOylation; ST rich denotes variable Ser/Thr-rich region. (**D**) HEK293T cells were co-transfected with HA-YTHDF2 and truncated versions of V5-PIAS1 or Halo-V5-PIAS1. WB analysis showing that YTHDF2 is Co-IPed with the N-terminal and middle part of PIAS1. β-Actin blot was included as loading control. (**E**) A proposed model showing the N- and C-terminal regions of YTHDF2 binding to the central part of PIAS1.

When YTHDF2 is IPed by anti-V5 beads, we noticed that PIAS1 is also co-immunoprecipitated (Co-IPed) with the beads (Fig. 1B; PIAS1 blot, lanes 3 and 4), indicating an interaction between PIAS1 and YTHDF2. To further determine whether YTHDF2 interacts with PIAS1 and which regions within YTHDF2 are responsible for this interaction, we transfected HEK293T cells with plasmids expressing full-length PIAS1 and full-length or individual fragments of YTHDF2 (Fig. 2). We then performed Co-IP experiments and found that PIAS1 is strongly Co-IPed by full-length YTHDF2 (Fig. 2B; top panel, lane 1). The Co-IP of PIAS1 was also observed in all YTHDF2 fragments except the central region of YTHDF2 (aa 167–367) (Fig. 2B; top panel, lanes 2, 3, 5, and 6 vs lane 4). These results suggested that both N-terminal (aa 1–166) and C-terminal (aa 368–579) of YTHDF2 bind to PIAS1.

To determine the region(s) within PIAS1 that interacts with YTHDF2, we co-transfected several truncated PIAS1 mutants with YTHDF2 into HEK293T cells (Fig. 2C). We then IPed YTHDF2 with anti-HA antibody-conjugated beads. We found that PIAS1 (aa 1–433), PIAS1 (aa 101–433), PIAS1 (aa 1–415), and PIAS1 (aa 1–205), but not PIAS1 (aa 409–651) and PIAS1 (aa 1–100), are Co-IPed by YTHDF2, suggesting that the central region of PIAS1 between SAP and RING domains (aa 101–205) is essential for YTHDF2 binding (Fig. 2D).

Together, our results indicated that both N-terminal and C-terminal parts of YTHDF2 interact with PIAS1, specifically with the middle region of PIAS1 (Fig. 2E).

### PIAS1 synergizes with YTHDF2 to inhibit EBV lytic replication

**Fig 3:**
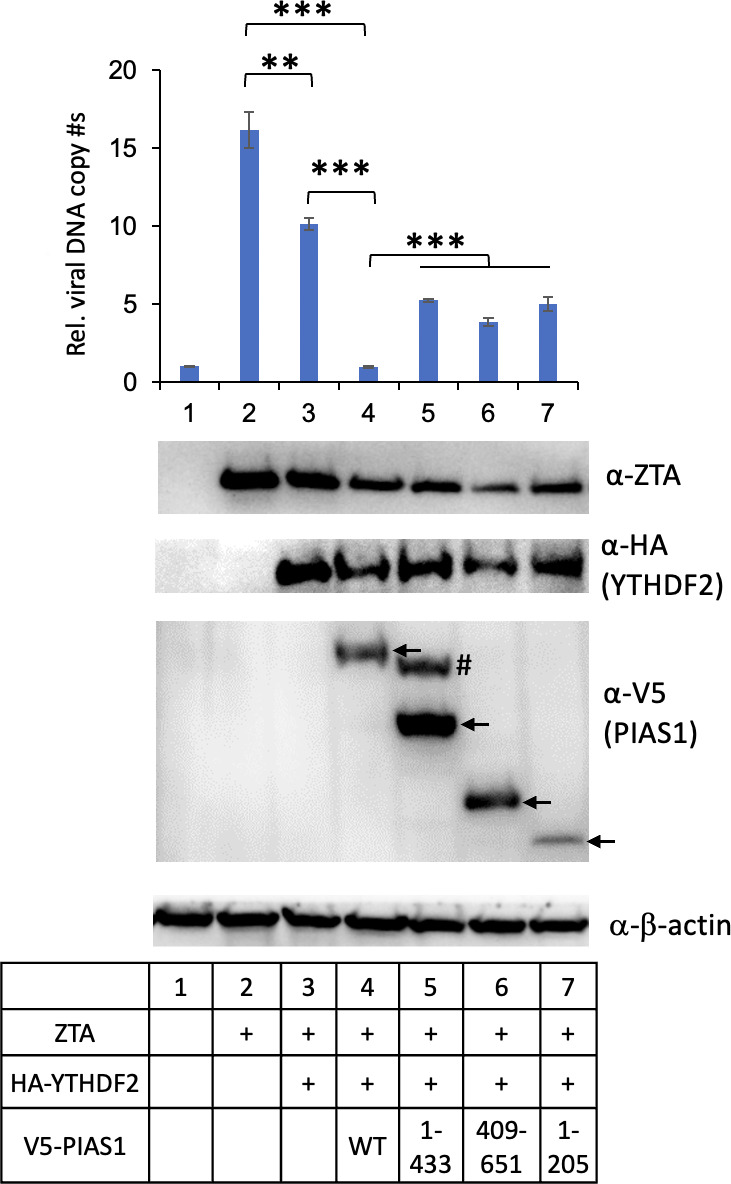
PIAS1 cooperates with YTHDF2 to suppress EBV lytic replication. HEK293 (EBV+) cells were co-transfected with plasmids encoding ZTA, YTHDF2, and truncated forms of PIAS1 (1–433, 409–651, and 1–205) as indicated. The relative EBV copy numbers were measured using the quantitative polymerase chain reaction (qPCR). The value of lane 1 was set as 1. The protein expression levels were monitored by WB using antibodies as indicated. β-Actin blot was included as loading control. Arrows denote the relative positions of PIAS1 fragments. # denotes a non-specific band. Results from three biological replicates are presented. Error bars indicate standard deviations. **P < 0.01, ***P < 0.001.

To investigate the potential function of PIAS1 interaction with YTHDF2, we performed co-transfection experiments. We transfected plasmids encoding ZTA (a trigger for EBV lytic reactivation), YTHDF2, and either full-length or truncated forms of PIAS1 into HEK293 (EBV+) cells. We observed that transfection of YTHDF2 alone led to a partial reduction in EBV lytic replication, while co-transfection of PIAS1 and YTHDF2 resulted in a significant reduction of EBV DNA replication (Fig. 3, lane 2 vs lane 3 and lane 3 vs lane 4). Interestingly, when truncated forms of PIAS1 were co-transfected, the enhanced anti-viral activity of YTHDF2 by PIAS1 was compromised, despite some of the fragments still exhibiting binding to YTHDF2 (Fig. 3, lane 4 vs lanes 5–7). Together, these results suggested that PIAS1 cooperates with YTHDF2 to limit EBV lytic replication.

### PIAS1 SUMOylates YTHDF2 at three major sites

**Fig 4:**
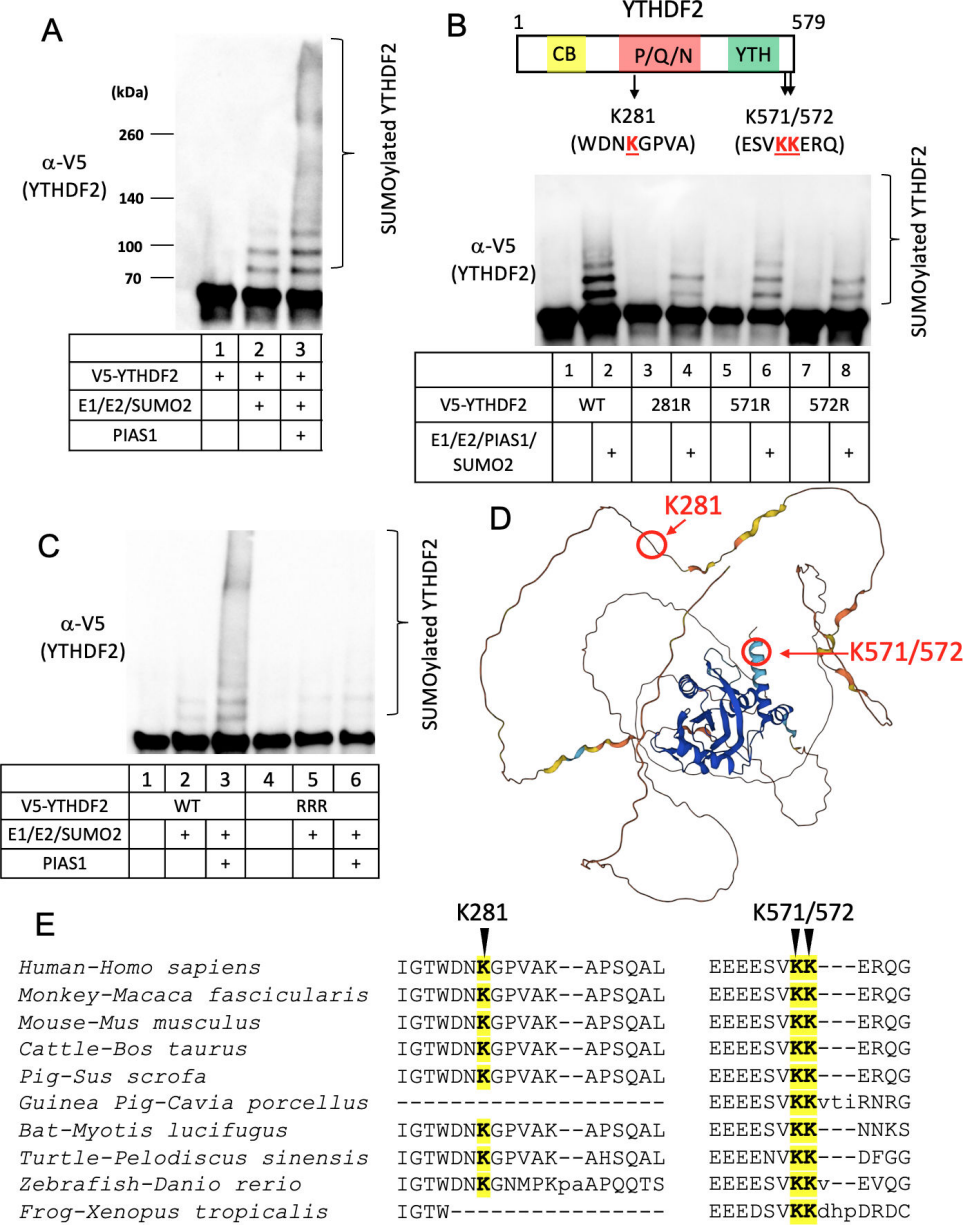
PIAS1 SUMOylates YTHDF2 at K281, K571, and K572. (**A**) *In vitro* SUMOylation assay was performed with the combination of purified E1, E2, SUMO2, PIAS1, and substrate V5-YTHDF2 proteins as indicated. The reaction was terminated with sodium dodecyl sulfate (SDS) sample loading buffer, and WB was performed using anti-V5-HRP antibody. Bracket denotes SUMOylated YTHDF2. (**B**) The schematic representation of YTHDF2 protein and the corresponding lysine residues (red) within SUMOylation consensus motifs. *In vitro* SUMOylation assay was performed with the combination of E1, E2, SUMO2, PIAS1, and either wild-type (WT) YTHDF2 or individual YTHDF2 mutants (K281R, K571R, and K572R) as indicated. The reaction was terminated with SDS sample loading buffer, and WB was performed using anti-V5-HRP antibody. (**C**) *In vitro* SUMOylation assay was performed for WT YTHDF2 and RRR mutant (K281R/K571R/K572R) as indicated. (**D**) Localization of K281, K571, and K572 in YTHDF2. The YTHDF2 three-dimensional structure was predicted by AlphaFold. The locations of indicated SUMOylation sites are marked by arrows. (**E**) Sequence alignment of YTHDF2 protein sequences from 10 species using the Constraint-based Multiple Alignment Tool (COBALT). The SUMOylation sites corresponding to human YTHFD2 are highlighted in yellow.

The strong interaction between PIAS1 and YTHDF2, along with the enhanced SUMOylation of YTHDF2 in the presence of PIAS1 (Fig. 1 and 2), supports the hypothesis that PIAS1 directly SUMOylates YTHDF2. To investigate this, we performed *in vitro* SUMOylation assays using recombinant proteins. We utilized purified V5-YTHDF2 protein and incubated it with E1, E2, SUMO2, and purified PIAS1 protein. Our results clearly demonstrated that PIAS1 significantly enhances the SUMOylation of YTHDF2 (Fig. 4A, lane 3 vs lanes 1 and 2).

Protein SUMOylation typically takes place on lysine residues within the consensus motif ΨKxE/D or the inverted motif E/DxKΨ, where Ψ represents a hydrophobic amino acid and x can be any amino acid residue. However, there are instances where SUMOylation occurs on lysine residues outside of the consensus sequence (17).

To identify the SUMOylation sites on YTHDF2, we searched for the consensus motif and identified two potential SUMOylation sites. The first site is K281, located within the DNKG sequence (DxKΨ), and the second site is K571, located within the VKKE sequence (ΨKxE) (Fig. 4B, top panel).

To demonstrate whether K281 and K571 can be SUMOylated by PIAS1, we created mutant YTHDF2 constructs in which each lysine residue was individually mutated to arginine. We also created a K572R mutant as it is located within the VKKE sequence. We purified these mutant proteins from HEK293T cells and performed *in vitro* SUMOylation assays.

Our results demonstrated that the SUMOylation level is reduced in all three mutant proteins (K281, K571, and K572) compared to the wild-type (WT) YTHDF2 (Fig. 4B; bottom panel, lanes 4, 6, and 8 vs lane 2), suggesting that all three sites can be SUMOylated even though K572 is not a consensus SUMOylation site. We then generated a mutant YTHDF2, K281R/K571R/K572R (RRR), in which all three lysine residues were simultaneously mutated to arginines. We found that the SUMOylation of YTHDF2 (RRR) mutant is abolished compared to WT protein (Fig. 4C, lane 6 vs lane 3). These findings together demonstrated that K281, K571, and K572 are the major SUMOylation sites on YTHDF2 mediated by PIAS1.

According to the YTHDF2 three-dimensional structure predicted by AlphaFold (18), we observed that K281 is situated within a disordered region, whereas K571 and K572 are located at the C-terminal end of YTHDF2, forming an alpha-helix secondary structure (Fig. 4D). The presence of a disordered region and the location of lysine residues at the very C-terminal region of YTHDF2 may contribute to structural flexibility favorable for SUMOylation.

To examine the conservation of YTHDF2 SUMOylation sites across different species, we performed an alignment of the amino acid sequence of human YTHDF2 with sequences from nine other species. Remarkably, we observed that the amino acids corresponding to K571/K572 of human YTHDF2 are conserved among all the examined species (Fig. 4E). As for K281 of human YTHDF2, we found that the corresponding sequences are highly conserved in human, monkey, mouse, cattle, pig, bat, turtle, and zebrafish, but are absent in guinea pig and xenopus YTHDF2 (Fig. 4E). This finding suggested a high likelihood of YTHDF2 SUMOylation by PIAS1 at the same positions in other organisms as observed in humans.

### YTHDF2 SUMOylation by PIAS1 significantly inhibits EBV replication

**Fig 5:**
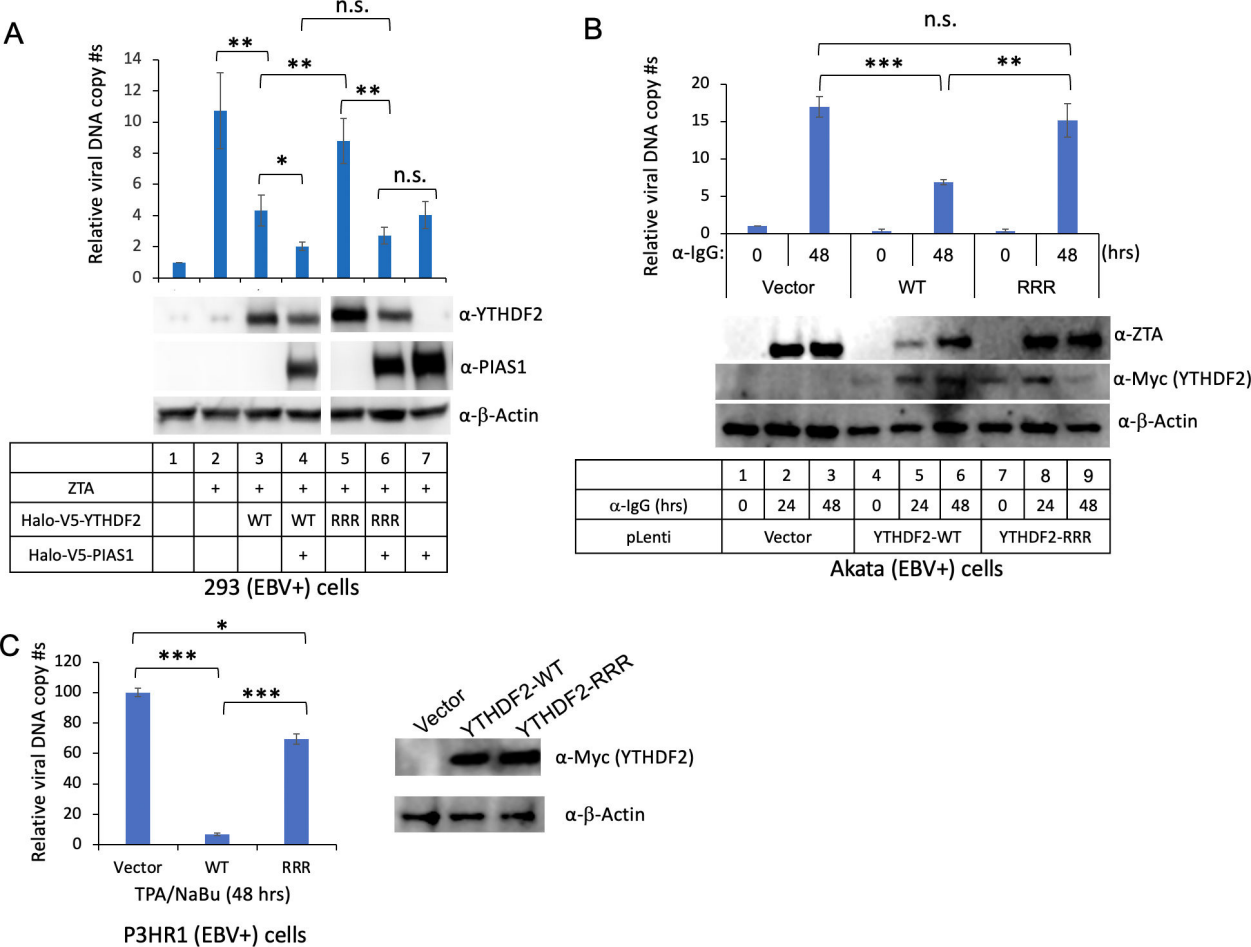
SUMOylation-deficient YTHDF2 impairs its ability to restrict EBV replication. (**A**) HEK293 (EBV+) cells were transfected with ZTA plasmid (lytic inducer), PIAS1, and WT YTHDF2 (WT) or SUMOylation-deficient YTHDF2 (RRR) as indicated. The relative EBV copy numbers were measured using the qPCR as described in the Materials and Methods. The value of lane 1 was set as 1. The expression levels of YTHDF2 and PIAS1 were monitored by WB. β-Actin blot was included as loading control. (**B**) Akata (EBV+) cells were used to create cell lines using pLenti-Vector, pLenti-YTHDF2-WT, and p-Lenti-YTHDF2-RRR (K281R/K571R/K572R). EBV lytic cycle was induced by anti-IgG-mediated B-cell receptor cross-linking. The relative EBV copy numbers were measured using the qPCR as described in Materials and Methods. The value of lane 1 was set as 1. The expression levels of ZTA and YTHDF2 were monitored by WB using anti-ZTA and anti-Myc antibodies, respectively. β-Actin blot was included as loading control. (**C**) P3HR1 (EBV+) cells were used to create cell lines using pLenti-Vector, pLenti-YTHDF2-WT, and p-Lenti-YTHDF2-RRR. EBV lytic cycle was induced by TPA and NaBu for 48 h. The relative EBV copy numbers were measured using the qPCR as described in Materials and Methods. The value of lane 1 was set as 100. The expression of YTHDF2 was monitored by WB using anti-Myc antibody. β-Actin blot was included as loading control. Results from three biological replicates are presented. Error bars indicate standard deviations. *P  <  0.05, **P  <  0.01, ***P  <  0.001. n.s., not significant; NaBu, sodium butyrate; TPA, tetradecanoyl phorbol acetate.

To determine the function of YTHDF2 SUMOylation in EBV replication, we introduced vectors expressing PIAS1, WT YTHDF2, and RRR mutant lacking SUMOylation sites. In the absence of PIAS1 co-transfection, the RRR mutant exhibited higher levels of EBV replication compared to WT YTHDF2 (Fig. 5A, lane 5 vs 3), suggesting a reduced anti-viral activity when the SUMOylation of YTHDF2 is blocked. However, when co-transfected with PIAS1, the SUMOylation-deficient mutant displays similar viral replication compared to cells expressing WT YTHDF2 (Fig. 5A, lane 6 vs lane 4), possibly because PIAS1 overexpression, together with endogenous PIAS1, also inhibits EBV replication (Fig. 5A, lane 7).

To demonstrate the synergistic effect of PIAS1 and YTHDF2, we depleted PIAS1 in HEK293 (EBV+) cells (Fig. S1A). We then transfected cells depleted of PIAS1, following a procedure similar to that of Fig. 5A. We found that, in the control cells, the regulation of YTHDF2 by PIAS1 in EBV replication is similar as that observed in the parental cells (Fig. S1B vs Fig. 5A). Notably, in PIAS1-depleted cells, PIAS1 synergized with WT YTHDF2 to limit EBV lytic replication (Fig. S1C, lane 3 vs lane 4). For the RRR mutant, the synergistic effect was largely diminished (Fig. S1C, lane 5 vs lane 6). These results suggested that PIAS1-mediated SUMOylation of YTHDF2 suppresses EBV lytic replication.

To further demonstrate the physiological relevance of YTHDF2 SUMOylation in EBV replication, we generated lentiviral constructs containing WT and RRR mutant YTHDF2. We transduced Akata (EBV+) Burkitt lymphoma cells with these constructs, establishing stable cell lines expressing either WT or RRR mutant YTHDF2. Upon lytic induction, we found a significant suppression of EBV DNA replication in cells expressing WT YTHDF2 (Fig. 5B, upper panel). However, we observed that cells expressing the RRR mutant exhibits higher levels of EBV replication compared to those expressing WT YTHDF2 (Fig. 5B, upper panel). Consistently, we found that viral protein ZTA expression is lower in WT YTHDF2-expressing cells than that in the RRR mutant cells (Fig. 5B; lower panel, lanes 5 and 6 vs lanes 8 and 9), suggesting that the loss of YTHDF2 SUMOylation abrogates its anti-viral activity.

To further confirm this observation, we also established WT and RRR mutant YTHDF2-expressing cell lines using EBV-positive P3HR1 Burkitt lymphoma cells. Similarly, we found that WT YTHDF2-expressing cells display much lower viral replication than those expressing the RRR mutant upon lytic induction (Fig. 5C). Together, these findings provided compelling evidence that PIAS1-mediated SUMOylation of YTHDF2 restricts EBV replication.

**Fig 6:**
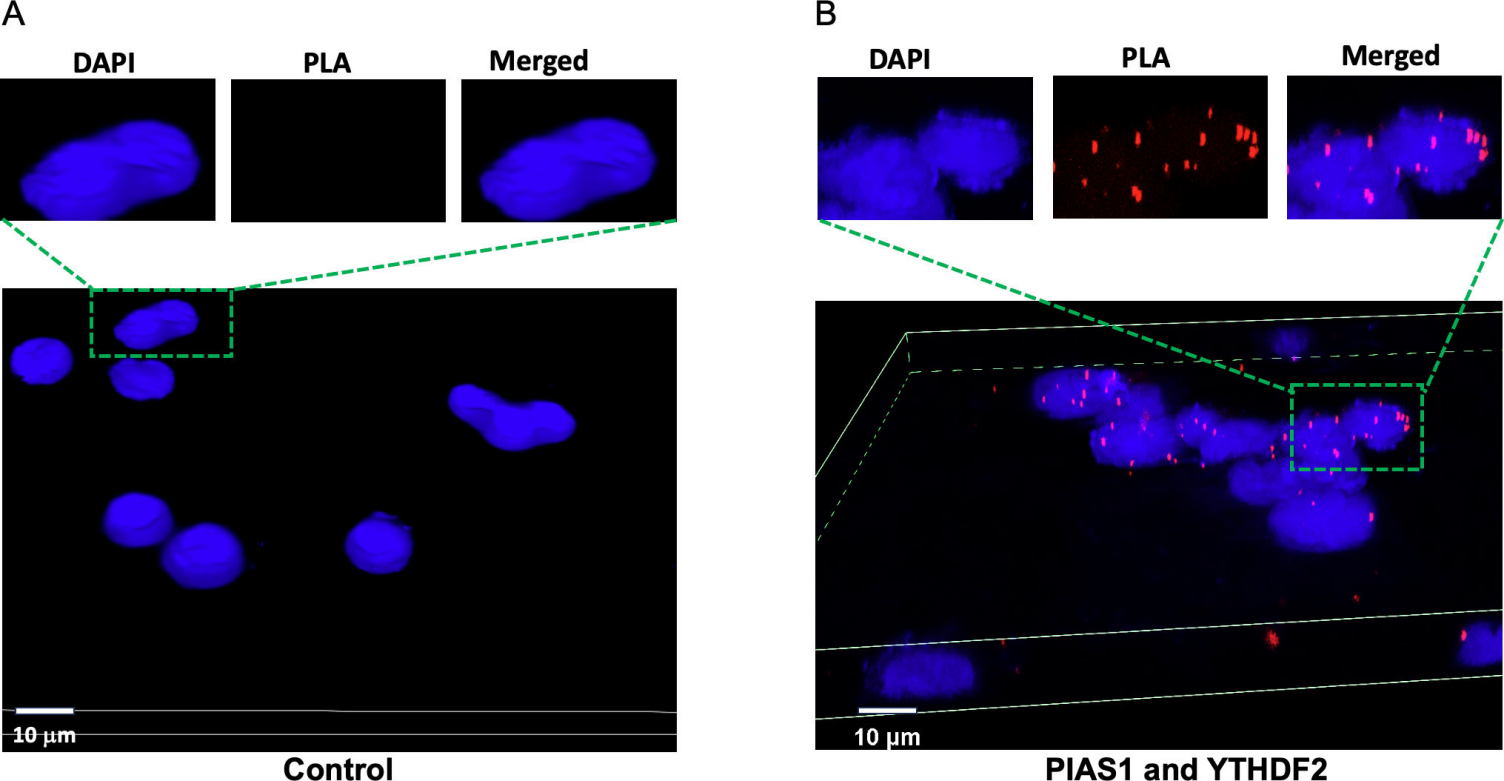
PIAS1 interacts with YTHDF2 *in situ*. Akata (EBV+) cells were blocked with 3% bovine serum albumin (BSA) in phosphate-buffered saline (PBS) at room temperature for 1 h, then incubated with PBS control (**A**) or a mixture of mouse anti-YTHDF2 and rabbit anti-PIAS1 antibodies (**B**). Then the probes were added for ligation and amplification. Cell nuclei were stained with DAPI and visualized using Nikon AXR. The red dots represent PLA signals indicating the interaction between YTHDF2 and PIAS1 *in situ*. DAPI, 4′,6-diamidino-2-phenylindole.

To further confirm the interaction between PIAS1 and YTHDF2 under physiological conditions, we conducted a proximity ligation assay (PLA) in Akata (EBV+) cells. Cells were subjected to PLA with or without the use of anti-PIAS1 and anti-YTHDF2 antibodies. Notably, in the absence of antibodies, no PLA signals were detected (Fig. 6A). In contrast, robust signals were observed in cells treated with anti-PIAS1 and anti-YTHDF2 antibodies for PLA, primarily localized in the nucleus (Fig. 6B). These findings strongly suggest that PIAS1 interacts with YTHDF2 and modifies its function through SUMOylation.

**Fig 7:**
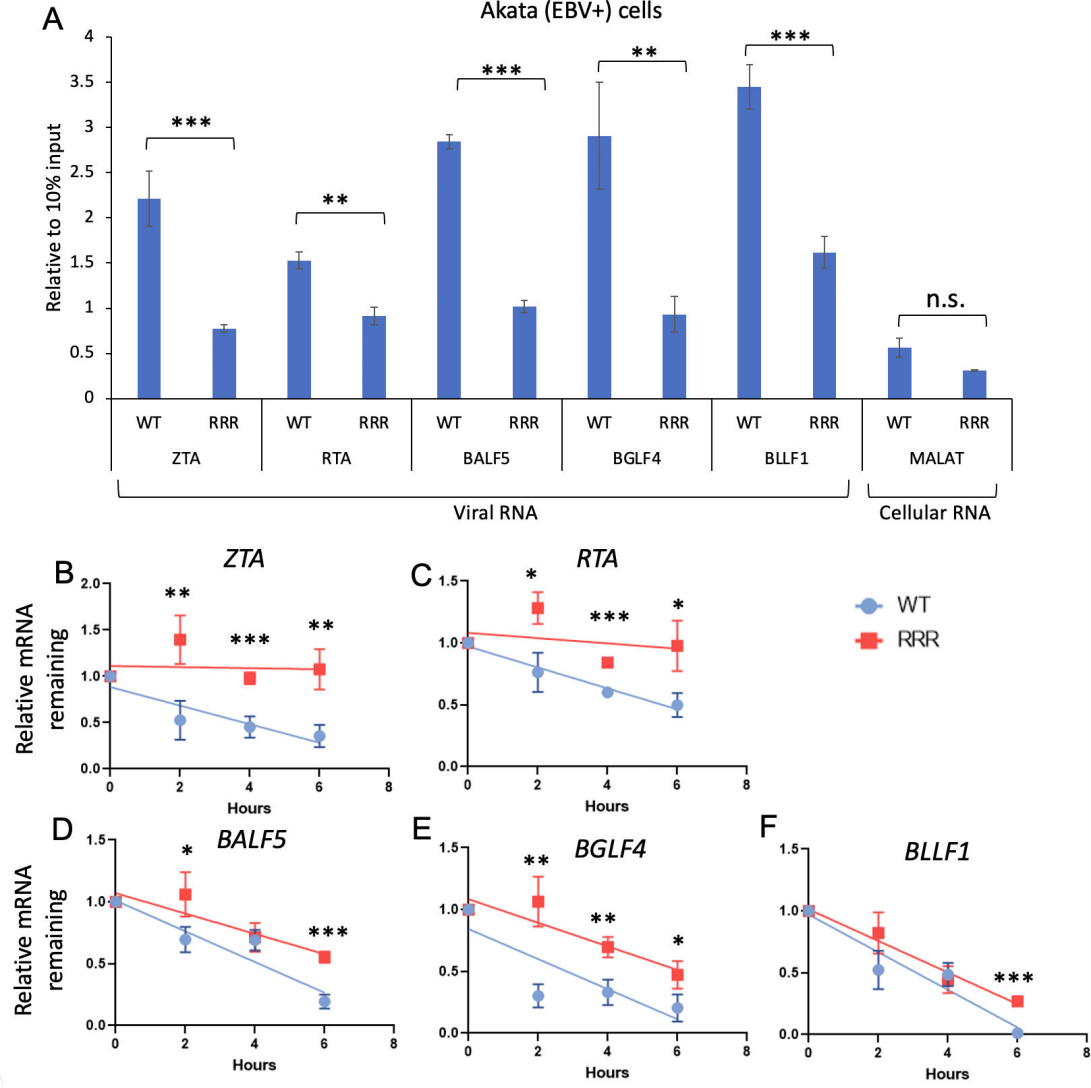
SUMOylation of YTHDF2 enhances its binding to and degradation of EBV lytic transcripts. Akata (EBV+) carrying WT YTHDF2 or SUMOylation-deficient mutant (RRR) were lytically induced by IgG cross-linking for 24 h. (**A**) Cell lysate was collected to detect YTHDF2 binding of viral RNAs by RIP-qPCR. Values are fold change over 10% input. Cellular RNA MALAT1 was included as a negative control. (**B–F**) After lytic induction for 12 h, the cells were then treated with actinomycin D. The immediate early (*ZTA* and *RTA*), early (*BALF5* and *BGLF4*), and late (*BLLF1*) gene levels were analyzed by quantitative reverse transcription-PCR. The relative mRNA level at 0 h after actinomycin D treatment was set as 1. Results from three biological replicates are presented. Error bars indicate standard deviations. *P < 0.05, **P < 0.01, ***P < 0.001. n.s., not significant.

SUMOylation of YTHDF2 has been implicated in binding to m6A modified RNAs (16). We reasoned that YTHDF2, after SUMOylation, affects its binding to viral lytic genes. To test this possibility, we performed YTHDF2 RNA immunoprecipitation (RIP) assay in Akata (EBV+) cells expressing WT or SUMOylation-deficient YTHDF2. Interestingly, we found that SUMOylation-deficient YTHDF2 displays reduced binding to EBV lytic transcripts compared to the WT counterpart (Fig. 7A).

YTHDF2 binding to viral RNA was shown to promote their degradation (5, 7). Therefore, the reduced binding seen in SUMOylation-deficient YTHDF2 may affect RNA stability. To test this idea, we monitored EBV lytic gene stability after actinomycin D treatment of WT or SUMOylation-deficient YTHDF2-expressing Akata (EBV+) cells preinduced with anti-IgG for either 12 or 24  h. We found that cells carrying SUMOylation-deficient YTHDF2 displays higher EBV lytic gene stability than cells with WT YTHDF2 (Fig. 7B through F and Fig. S2).

Together, these results suggested that SUMOylated YTHDF2 has higher binding affinity to EBV lytic transcripts, therefore facilitating their decay to restrict EBV lytic replication.

### PIAS1 facilitates the SUMOylation of YTHDF1 and YTHDF3

**Fig 8:**
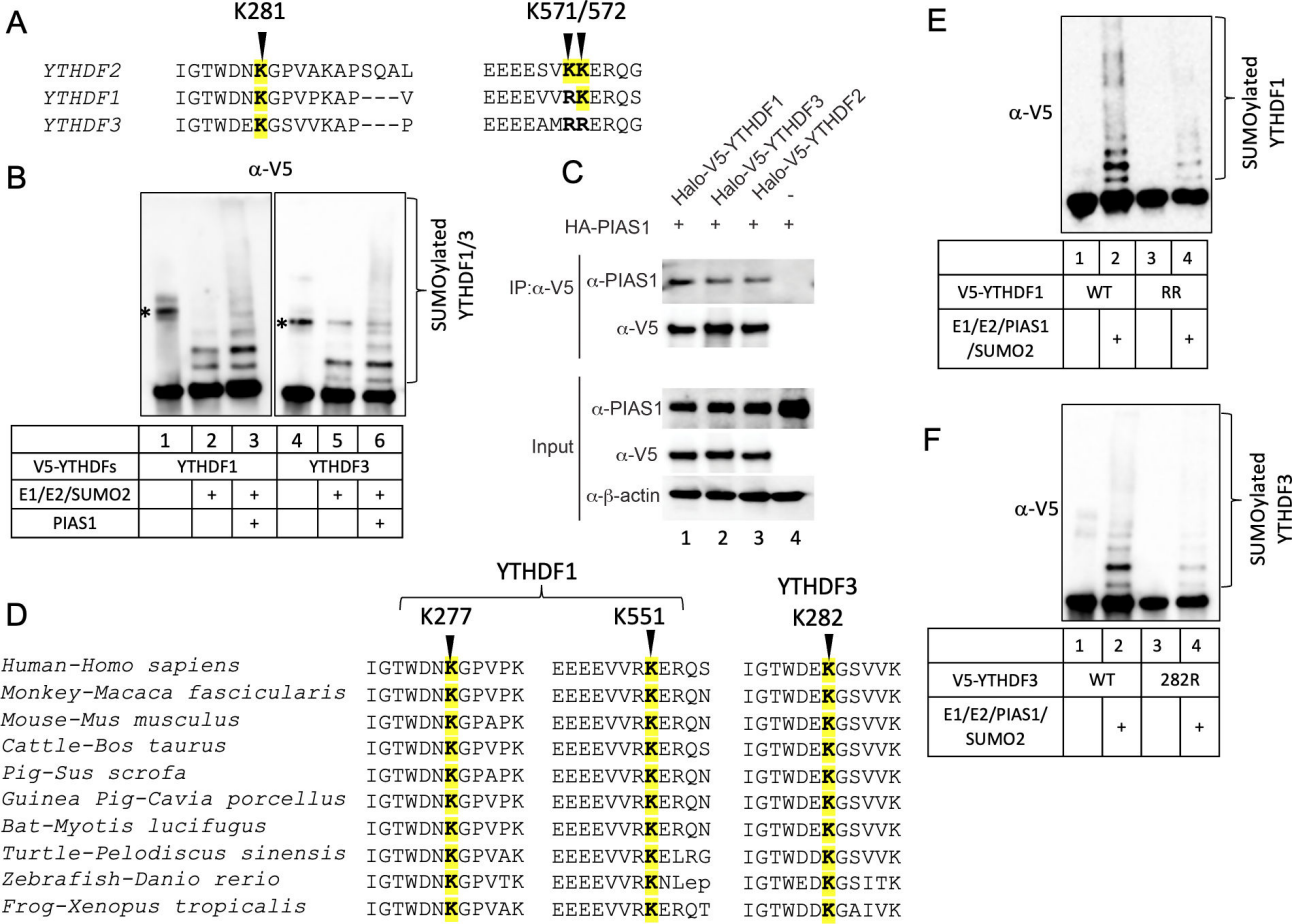
YTHDF1 and YTHDF3 are also SUMOylated by PIAS1. (**A**) Sequence alignment of human YTHDF2, YTHDF1 and YTHDF3 using COBALT. The SUMOylation sites corresponding to human YTHDF2 are highlighted in yellow. (**B**) PIAS1 promotes YTHDF1 and YTHDF3 SUMOylation. *In vitro* SUMOylation of YTHDF1 and YTHDF3 was performed with E1, E2, SUMO2 and PIAS1 as indicated. The reaction was terminated with SDS sample loading buffer, and WB was perfromed using anti-V5-HRP antibody. *, non-specific band. (**C**) PIAS1 interacts with YTHDF1 and YTHDF3. HEK293T cells were co-transfected with HA-PIAS1 and V5-YTHDF1, V5-YTHDF3, as well as V5-YTHDF2 as indicated. WB analysis showing that PIAS1 is Co-IPed with YTHDF1 and YTHDF3. Whole-cell lysates were blotted for PIAS1 and V5-YTHDFs as input. β-Actin blot was included as loading control. (**D**) Sequence alignment of YTHDF1 and YTHDF3 protein sequences from 10 species using COBALT. The positions of lysine corresponding to human YTHDF1 and YTHDF3 were labeled as indicated. The conserved lysines are highlighted in yellow. (**E**) *In vitro* SUMOylation assay was performed with the combination of E1, E2, SUMO2, PIAS1, and either WT or K277R/K551R mutant (RR) YTHDF1. The reaction was terminated with SDS sample loading buffer, and WB was performed using anti-V5-HRP antibody. (**F**) *In vitro* SUMOylation assay was performed with the combination of E1, E2, SUMO2, PIAS1, and either WT or K282R mutant YTHDF3. The reaction was terminated with SDS sample loading buffer and WB was performed using anti-V5-HRP antibody. COBALT, Constraint-based Multiple Alignment Tool.

YTHDF1 and YTHDF3 are paralogs of YTHDF2, and they exhibit redundant functions in regulating mRNA degradation within cells (19). Notably, we and others have demonstrated that both YTHDF1 and YTHDF3 play a role in restricting EBV infection (7, 20). By aligning the amino acid sequences of YTHDF1 and YTHDF3 with that of YTHDF2, we observed that the lysine corresponding to YTHDF2 K281 is highly conserved in both YTHDF1 and YTHDF3. Additionally, while YTHDF2 K571 is not conserved in YTHDF1 and YTHDF3, YTHDF1 does show conservation of the lysine corresponding to YTHDF2 K572 (Fig. 8A).

Based on this observation, we hypothesized that YTHDF1 and YTHDF3 can undergo SUMOylation and that PIAS1 might promote their SUMOylation. To test this hypothesis, we performed *in vitro* SUMOylation assays using purified YTHDF1 and YTHDF3, along with PIAS1 and the necessary enzymes and proteins (E1, E2, and SUMO2). The results from WB analysis demonstrated that YTHDF1 and YTHDF3 are SUMOylated, and the presence of PIAS1 enhances the SUMOylation of YTHDF1 and, to a lesser extent, YTHDF3 (Fig. 8B, lane 3 vs lanes 1 and 2 and lane 6 vs lanes 4 and 5).

To investigate the interaction between YTHDF1, YTHDF3, and PIAS1, we transfected HEK293T cells with plasmids expressing HA-PIAS1, V5-YTHDF1, V5-YTHDF3, and V5-YTHDF2 as a control. Following immunoprecipitation of YTHDF proteins using antibodies against V5 tag, we observed a robust interaction between PIAS1 and both YTHDF1 and YTHDF3, similar to the interaction observed with YTHDF2 (Fig. 8C, lanes 1–2 vs lane 3).

We analyzed the sequence conservation of YTHDF1 and YTHDF3 with nine other species. The analysis revealed that the lysines corresponding to K277 and K551 in human YTHDF1, as well as K282 in human YTHDF3, are conserved across all the examined species (Fig. 8D).

To demonstrate whether K277 and K551 in YTHDF1 and K282 in YTHDF3 can be SUMOylated by PIAS1, we created mutant constructs in which lysine residues were mutated to arginines. We purified these mutant proteins from HEK293T cells and performed *in vitro* SUMOylation assays.

We found that the SUMOylation levels of YTHDF1 (K277R/K551R) and YTHDF3 (K282R) are greatly reduced compared to their WT counterparts (Fig. 8E and F). These findings together demonstrated that K277 and K551 in YTHDF1 and K282 in YTHDF3 are the major SUMOylation sites mediated by PIAS1. The residual SUMOylation in these mutants also suggested other sites could be SUMOylated.

**Fig 9:**
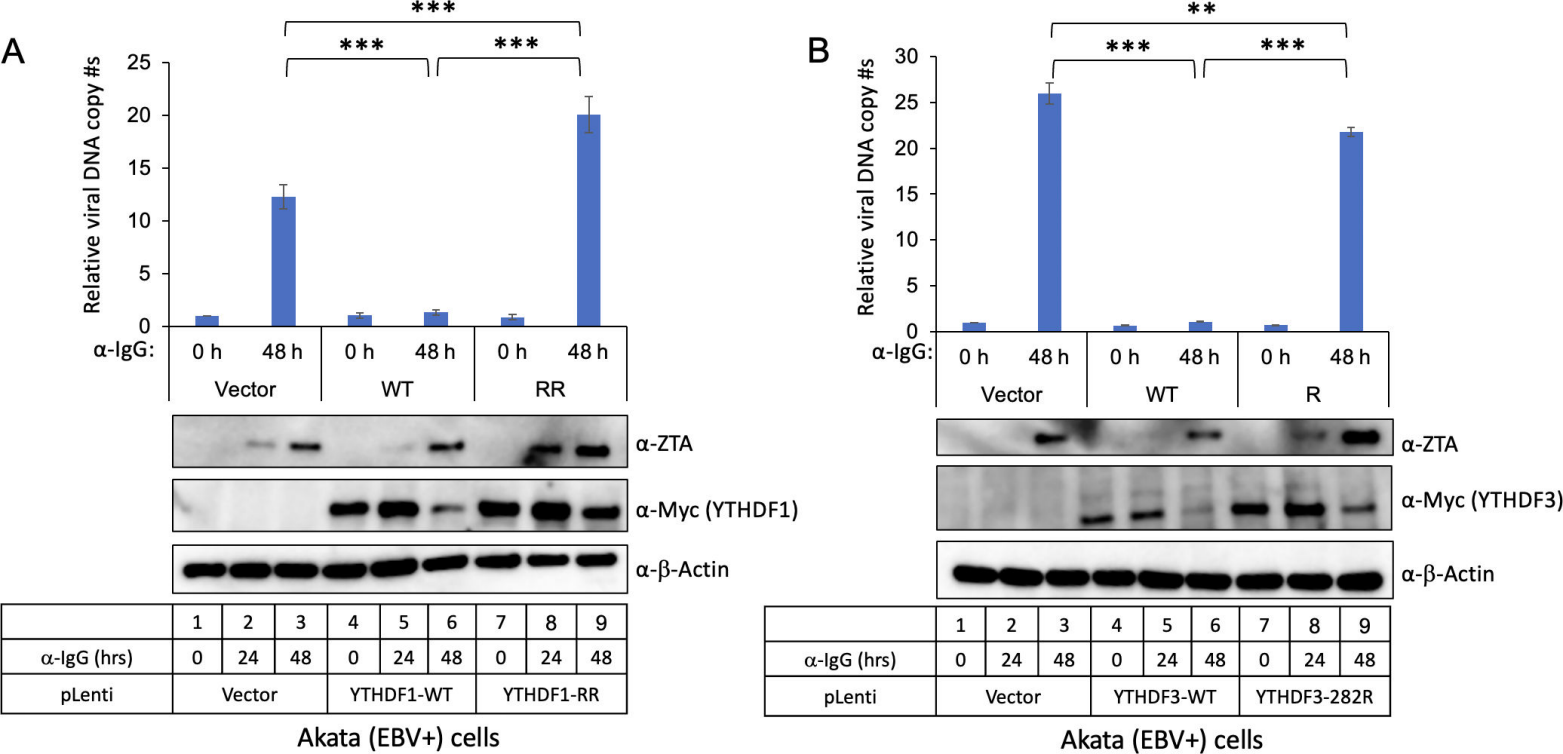
PIAS1-mediated SUMOylation of YTHDF1 and YTHDF3 restrict EBV lytic replication. (**A**) Akata (EBV+) cells were used to create cell lines using pLenti-Vector, pLenti-YTHDF1-WT, and p-Lenti-YTHDF1-RR (K277R/K551R). (**B**) Akata (EBV+) cells were used to create cell lines using pLenti-Vector, pLenti-YTHDF3-WT, and p-Lenti-YTHDF3-R (K282R). EBV lytic cycle was induced by anti-IgG-mediated B-cell receptor cross-linking. The relative EBV copy numbers were measured using the qPCR as described in Material and Methods. The value of lane 1 was set as 1. The expression levels of ZTA and YTHDF proteins were monitored by WB using anti-ZTA and anti-Myc antibodies, respectively. β-Actin blot was included as loading control. Results from three biological replicates are presented. Error bars indicate standard deviations. **P < 0.01, ***P < 0.001.

The presence of conserved residues in these regions implies that the SUMOylation of YTHDF1 and YTHDF3, as well as their targeting by PIAS1, plays an important role in controlling their function. To test this hypothesis, we created lentiviral constructs containing WT YTHDF1, YTHDF1 (K277R/K551R), WT YTHDF3, and YTHDF3 (K282R). We transduced Akata (EBV+) Burkitt lymphoma cells with these constructs, establishing stable cell lines expressing either WT or mutant YTHDF1/3. Upon lytic induction, we found a significant suppression of EBV DNA replication in cells expressing WT YTHDF1 (Fig. 9A). However, we observed that cells expressing YTHDF1 (K277R/K551R) exhibit higher levels of EBV replication compared to those expressing WT YTHDF1 (Fig. 9A). Similarly, cells expressing WT YTHDF3, but not YTHDF3 (K282R), displayed lower EBV replication upon lytic induction (Fig. 9B). These results together suggest that the loss of YTHDF1 and YTHDF3 SUMOylation abrogates their ability to suppress EBV lytic replication.

## DISCUSSION

The m6A RNA modification pathway has been implicated in a variety of cellular process by controlling RNA stability, splicing, and translation (2, 3, 21). This pathway is regulated by a group of cellular proteins that act as “writers,” “erasers,” and “readers” of the m6A mark. METTL3, METTL14, WTAP, VIRMA, and other associated proteins function as writers to methylate the specific adenosines in the RNA molecules. FTO and ALKBH5 function as erasers to remove the methyl group, thereby reversing the modification. The readers, including YTHDF1/YTHDF2/YTHDF3, YTHDC1/YTHDC2, and HNRNPA2B1, are responsible for binding to the m6A-modified RNA to regulate RNA metabolism (2). Recently, the m6A RNA modification pathway has been studied for its role in the life cycle of a variety of viruses, including herpesviruses (21–23). M6A modification and YTHDF2 reader have been shown to regulate KSHV RNA stability and reactivation (4, 5).

We and others have shown that the m6A writers (METTL3, METTL14, WTAP, and VIRMA) and readers (YTHDF1, YTHDF2, and YTHDF3) restrict EBV reactivation (6, 7, 20, 24). On the other hand, several studies suggested that the m6A RNA modification enzymes and readers regulate interferon production to control the infection of KSHV, EBV, human cytomegalovirus, vaccinia virus, and herpes simplex virus type 1 (HSV-1) (25–30).

PTMs, such as ubiquitination, SUMOylation, phosphorylation, methylation, acetylation, and proteolytic cleavage, play crucial roles in determining the fate and function of proteins. Several members of the m6A RNA modification pathway undergo PTMs. We recently have shown that caspase-mediated cleavage of the m6A pathway writers and readers promotes viral replication (7). Phosphorylation has been implicated in the regulation of the m6A methyltransferase complex (31). Although WTAP and METTL3 phosphorylation has been shown to enhance the activity of the methyltransferase complex through protein stabilization (32), alphaherpesvirus pseudorabies virus, and HSV-1 encoded protein kinases US3 inactivate the writer complex through phosphorylation (33). While YTHDF2 has been shown to be degraded via the ubiquitin-proteasome pathway when CDK1 is activated (10), phosphorylation of YTHDF2 by EGFR/SRC/ERK signaling prevents its degradation (9).

The SUMOylation of METTL3 has been demonstrated to slightly suppress its methyltransferase activity (34). ALKBH5 is SUMOylated by PIAS4 to inhibit its m6A demethylase activity by blocking substrate accessibility (35). Additionally, YTHDF2 has also been identified as a target for SUMOylation, which enhances its binding affinity to m6A-modified RNAs to promote RNA degradation (16).

In this study, we demonstrated for the first time that YTHDF2 is SUMOylated by the E3 SUMO ligase PIAS1 (Fig. 1 and 4). We observed that both the N-terminal and C-terminal regions of YTHDF2 are involved in the interaction with PIAS1. Similarly, the central part of PIAS1 was found to interact with YTHDF2 (Fig. 2). The strong interaction between YTHDF2 and PIAS1 was further verified by robust signals detected by PLA (Fig. 6), suggesting that PIAS1 may regulate the anti-viral activity of YTHDF2. Indeed, we found that the inhibition of EBV replication by YTHDF2 was significantly enhanced in the presence of full-length PIAS1, but less so in the presence of truncated PIAS1 mutants (Fig. 3). These results suggested that the regulation of YTHDF2 SUMOylation mediated by PIAS1 contributes to its anti-viral activity, like our previous observations with SAMHD1 (14).

During our investigation into the SUMOylation of YTHDF2, we remained open-minded regarding the potential presence of additional SUMOylation sites, despite the identification of K571 as one modification site in a previous study (16). Interestingly, we uncovered two novel SUMOylation sites in YTHDF2, namely, K281 and K572 (Fig. 4).

The discovery of K281 as a SUMOylation site in YTHDF2 highlights a modification occurring within the P/Q/N disordered region, which is highly conserved among YTHDF2 homologs from various species (Fig. 4). The identification of K572 reveals the presence of a non-consensus SUMOylation site that may have a redundant role in YTHDF2 SUMOylation, which further supports the notion that non-consensus SUMOylation is a common event in cells (17, 36).

To investigate the role of YTHDF2 SUMOylation in EBV replication, we generated a SUMOylation-deficient YTHDF2 mutant. Our findings revealed SUMOylation-deficient YTHDF2 displayed reduced anti-viral activity due to its reduced binding to and degradation of viral RNAs (Fig. 7) (16). Moreover, the establishment of stable cell lines expressing WT or mutant YTHDF2 further stressed the importance of SUMOylation in suppressing EBV lytic replication in multiple EBV-infected tumor cells (Fig. 5). Notably, previous studies have demonstrated that YTHDF2 undergoes ubiquitination (10, 30). Therefore, SUMOylation could potentially hinder ubiquitination and subsequent degradation of YTHDF2, which requires further investigation.

The sequence conservation between YTHDF2 and its paralogs led us to investigate the SUMOylation of YTHDF1 and YTHDF3 by PIAS1 (Fig. 8). We found that the amino acids corresponding to K281 in YTHDF2 (namely K277 in YTHDF1 and K282 in YTHDF3) and K572 in YTHDF2 (namely K551 in YTHDF1) are conserved across all ten species examined. Consistently, the mutation of these sites reduced their SUMOylation level by PIAS1 and compromise their capability to inhibit EBV lytic replication (Fig. 9). The residual SUMOylation suggested additional sites could be SUMOylated. PIAS1 interacts with all members of the YTHDF family. Interestingly, structure prediction by a recently developed AlphaFold-Multimer algorithm (37) suggested that the PIAS1 aa 161–239 region interacts with the YTH domain of YTHDF1, YTHDF2, and YTHDF3 (Fig. S3). This is consistent with our Co-IP results showing that the central part of PIAS1 (aa 101–433) interacts with YTHDF2 (Fig. 2).

**Fig 10:**
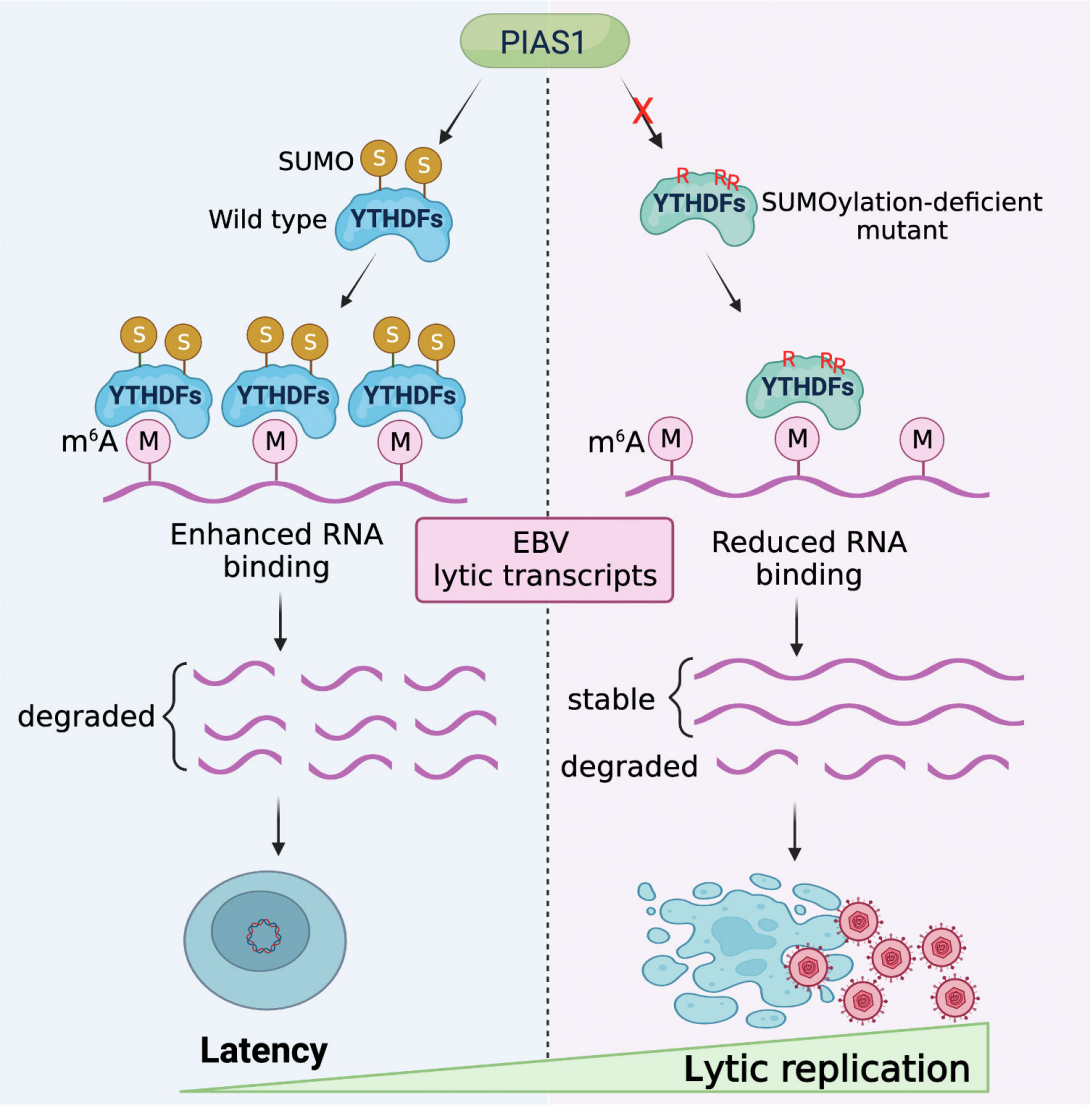
Model summarizing PIAS1-mediated SUMOylation of YTHDF family proteins in control of EBV latency and reactivation.

In conclusion, our findings demonstrated that YTHDF2 and its paralogs, YTHDF1 and YTHDF3, undergo SUMOylation at conserved lysine residues, and PIAS1 plays a crucial role in this process to restrict EBV lytic replication (Fig. 10). Our observations provided insights into the conservation and functional significance of PIAS1-mediated SUMOylation of YTHDF2, as well as YTHDF1 and YTHDF3, suggesting that SUMOylation may play a role in regulating the activities of these proteins in RNA metabolism and consequently other cellular processes. METTL3, as demonstrated by Du et al. in 2018, has already been identified as a target for regulation through SUMOylation (34). It will be interesting to explore whether METTL3 and other members of the m6A RNA modification pathway are regulated by PIAS1-mediated SUMOylation and the associated impacts on RNA modification, viral infection, and host defense.

## MATERIALS AND METHODS

### Cell lines and cultures

Akata (EBV+) and P3HR-1 cells were cultured in Roswell Park Memorial Institute medium (RPMI 1640) supplemented with 10% FBS (Cat #26140079, Thermo Fisher Scientific) in 5% CO_2_ at 37°C (13, 38–41). HEK293 (EBV+) cells with B95.8 EBV genome were provided by Delecluse et al. and El-Guindy et al. (42, 43). HEK293 (EBV+), and 293T cells were cultured in Dulbecco’s modified eagle medium supplemented with 10% FBS in 5% CO_2_ at 37°C.

### Plasmid construction

Halo-PIAS1, Halo-V5-PIAS1 (full length and aa 1–100), and V5-PIAS1 (full length, aa 1–205, aa 1–415, aa 1–433, aa 409–651, and aa 101–433) plasmids were previously described (13, 15). Halo-V5-YTHDF2, (full length, aa 1–166, aa 1–367, aa 167–367, aa 167–579, and aa 368–579) plasmids were constructed by our lab previously (7). Halo-V5-YTHDF2 was used as a template to create K281R, K571R, and K572R mutants using the QuikChange II site-directed mutagenesis kit (Cat #200521, Stratagene) according to the manufacturer’s instructions.

Halo-V5-YTHDF1 and Halo-V5-YTHDF3 were constructed using methods described previously (7). These plasmids were then used to create Halo-V5-YTHDF1 (K277R/K552R) and Halo-V5-YTHDF3 (K282R).

Halo-V5-YTHDF2-K281R/K571R/K572R, Halo-V5-YTHDF1-K277R/K551R, and Halo-V5-YTHDF3-K282R were created using QuikChange II site-directed mutagenesis kit step by step. The pLenti-C-Myc-DDK-P2A-BSD and pCMV6-Entry-YTHDF2 were purchased from Origene. The specific variants were generated by site-directed mutagenesis in pCMV6-Entry-YTHDF2 using the QuikChange II site-directed mutagenesis kit. Subsequently, the WT and mutated YTHDF2 in the pCMV6-Entry vector and the PCR amplicons of WT and mutated YTHDF1 and YTHDF3 from Halo-V5-YTHDF1 and Halo-V5-YTHDF3 were digested using AsiSI and MluI and then subcloned into the pLenti-C-Myc-DDK-P2A-BSD vector. All primer sequences are listed in Table S1.

### *In situ* PLA

Akata (EBV+) cells were fixed with cold methanol for 5 min at −20°C followed by permeabilization with 0.5% Triton X-100 for 5 min. The PLA experiments were performed using Duolink *in situ* PLA kit (DUO92101, Sigma) according to manufacturer’s instructions. Briefly, cells were blocked with 3% BSA in PBS at room temperature for 1 h, then incubated with PBS control or a mixture of mouse anti-YTHDF2 (H00051441-B01P, Abnova) and rabbit anti-PIAS1 (ab77231, Abcam) antibodies (1:500 dilution in PBS) at 4°C overnight. Then the probes were incubated at 37°C for 1 h, followed by ligation and amplification. Cell nuclei were stained using Duolink *in situ* mounting media with 4′,6-diamidino-2-phenylindole and visualized by Nikon AXR confocal microscope.

### Lentiviral transduction

Lentiviruses were prepared using 293T cells transfected with lentiviral vector containing the target gene and the helper vectors (pMD2.G and psPAX2) as previously descripted (13, 44). Akata (EBV+) and P3HR-1 cells were transduced with lentiviruses carrying vector control, WT and mutants YTHDF2, YTHDF1, and YTHDF3 to establish cell lines in RPMI medium containing 10-µg/mL blasticidin. HEK-293 (EBV+) was transduced with lentivirus carrying CRISPR/Cas9 PIAS1-sg1 or sg-NC (13) and selected in a medium containing 2-µg/mL puromycin.

### Cell lysis, immunoprecipitation, and immunoblotting

Cells lysis, immunoprecipitation, and immunoblotting (WB) were performed as previously described (14).

### Protein expression and purification

Halo-tagged PIAS1, YTHDF2, YTHDF1, and YTHDF3 proteins were expressed and purified as previously described (13, 40, 44).

### *In vitro* SUMOylation assay

*In vitro* SUMOylation assay was performed using the SUMO2 conjugation kit. The assay was conducted in a buffer comprising 40-mM Tris, pH 7.1, 40-mM NaCl, 1-mM β-mercaptoethanol, and 5-mM MgCl_2_. Each protein SUMOylation reaction included 100-nM SAE1/SAE2 (E1), 2-µM 6xHis-Ube2I/UBC9 (E2), 50-µM SUMO2, and 4-mM ATP. Additionally, purified PIAS1 was introduced as the E3 ligase. The reaction mixtures were then incubated at 37°C for 3 h, and WB analysis was carried out to examine the SUMOylation of YTHDF2, YTHDF1, and YTHDF3 proteins.

### Lytic induction

Akata (EBV+) cells were treated with IgG (1:200, Cat #55087; MP Biomedicals) to induce lytic replication for up to 48 h. To induce the EBV lytic cycle in P3HR-1 cells, the cells were triggered with tetradecanoyl phorbol acetate (20 ng/mL) and sodium butyrate (3  mM) for up to 48 h (7). For lytic induction of EBV in HEK293 (EBV+) cells, the cells were transfected with EBV ZTA plus other plasmids as appropriate using Lipofectamine 2000 reagent or PEI max for 48 h (Table 1).

**TABLE 1:** Key reagents and resources

Reagent or resource	Source	Identifier
Antibodies and reagents		
Anti-PIAS1	Cell Signaling Tech	Cat #15038
Anti-PIAS1	Abcam	Cat #ab77231
Anti-YTHDF2	Bethyl	Cat #A311-354
Anti-YTHDF2	Abnova	Cat #H00051441-B01P
Anti-Myc	Cell Signaling Tech	Cat #2278
Anti-c-Myc magnetic beads	Thermo Scientific	Cat #88842
Anti-HA	Roche	Cat #11–867-431-001
Anti-HA-HRP	Cell Signaling Tech	Cat #14031
SUMO2 conjugation kit	UBPBio	Cat #J3120
Anti-V5 magnetic beads	MBL	Cat #M167-11
Anti-SUMO2/3	MBL	Cat #M114-3
Anti-SUMO2/3	Active motif	Cat #101898
Anti-V5-HRP	Thermo Fisher	Cat #R961-25
Anit-V5	Thermo Fisher	Cat #R960-25
Mouse anti-β-actin antibody	MP Biomedicals	Cat #91001
Anti-human IgG (for IgG cross-linking)	MP Biomedicals	Cat #55087
Anti-ZTA(BZ1)	Santa Cruz	Cat #sc-53904
Halo-tag protein purification kit	VWR/Promega	Cat #PAG6790
Tetradecanoyl phorbol acetate	Fisher Scientific	Cat #NC9325685
Sodium Butyrate	Millipore	Cat #9137
Actinomycin D	Sigma	Cat #A1410
Magna RIP RNA-binding protein immunoprecipitation kit	Millipore	Cat #17–700
Genomic DNA Purification Kit	Promega	Cat #A1120
Isolate II RNA minikit	Bioline	Cat #BIO-52073
Lipofectamine 2000	Life Technologies	Cat #11668019
PEI Max	Polysciences	Cat#24765-100
Constructs		
pHalo-V5-YTHDF2	Li Lab (7)	pKZ157
pHalo-V5-YTHDF2-1-166	Li Lab (7)	pKZ220
pHalo-V5-YTHDF2-1-367	Li Lab (7)	pKZ221
pHalo-V5-YTHDF2-167-367	Li Lab (7)	pKZ222
pHalo-V5-YTHDF2-167-579	Li Lab (7)	pKZ227
pHalo-V5-YTHDF2-368-579	Li Lab (7)	pKZ228
pCMV6-Entry-YTHDF2	Origene	Cat #RC200038
pLenti-C-Myc-DDK-P2A-BSD (vector)	Origene	Cat #PS100103
pLenti-C-Myc-YTHDF2 (with PAM mutated)	Li Lab (7)	pKZ249
Halo-V5-YTHDF2-K571R	This study	pSF020
Halo-V5-YTHDF2-K572R	This study	pSF042
Halo-V5-YTHDF2-K281R	This study	pSF045
Halo-V5-YTHDF2- K281R/K571R/K572R (RRR)	This study	pSF149
pCMV6-Enrty-YTHDF2- K281R/K571R/K572R (RRR)	This study	pSF151
p-lenti- C-Myc-YTHDF2	Li Lab (7)	pKZ243
p-lenti- C-Myc-YTHDF2-K281R/K571R/K572R (RRR)	This study	pSF160
p-lenti- C-Myc-YTHDF1	This study	pFS338
p-lenti- C-Myc-YTHDF1-K277R/K551R (RR)	This study	pFS342
p-lenti- C-Myc-YTHDF3	This study	pFS323
p-lenti- C-Myc-YTHDF3-K282R (R)	This study	pFS343
Halo-V5-YTHDF1	This study	pFS52
Halo-V5-YTHDF3	This study	pFS53
Halo-V5-YTHDF1-K277R/K571R (RR)	This study	pFS339
Halo-V5-YTHDF3-K282R	This study	pFS337
pMD2.G	Addgene	Plasmid #12259
psPAX2	Addgene	Plasmid #12260
pSG5-ZTA	Hayward Lab Collection	NA
Halo-V5-PIAS1	Li Lab (13)	pKZ28
Halo-PIAS1	Li Lab (13)	pKZ27
His-SUMO2	(17)	NA
V5-PIAS1	Li Lab (13)	pKZ6b
V5-PIAS1 (1-415)	Li Lab (13)	pKZ15
V5-PIAS1 (409-651)	Li Lab (13)	pKZ16
V5-PIAS1 (1-433)	Li Lab (13)	pKZ103
V5-PIAS1 (101-433)	Li Lab (13)	pKZ83
V5-PIAS1 (1-205)	Li Lab (15)	pKZ148
pHalo-V5-PIAS1 (1-100)	Li Lab (15)	pKZ85
Cell lines		
Akata (EBV+)	Hayward Lab Collection	NA^*a*^
Akata (EBV+)-pLenti-Vector	This study	NA
Akata (EBV+)-pLenti-YTHDF2	This study	NA
Akata (EBV+)-pLenti-YTHDF2-K281R/K571R/K572R (RRR)	This study	NA
Akata (EBV+)-pLenti-YTHDF1	This study	NA
Akata (EBV+)-pLenti-YTHDF1-K277R/K551R (RR)	This study	NA
Akata (EBV+)-pLenti-YTHDF3	This study	NA
Akata (EBV+)-pLenti-YTHDF3-K282R (R)	This study	NA
293T cells	Hayward Lab Collection	NA
HEK 293 (EBV+)	(42)	NA
P3HR1	Hayward Lab Collection	NA

^*a*^ NA, not available.

### EBV copy number detection

To extract cell associated viral DNA, total genomic DNA was extracted using the Genomic DNA Purification Kit (Cat #A1120, Promega). The relative viral genome copy numbers were determined by quantitative polymerase chain reaction (qPCR) using primers specific to *BALF5* gene normalized by β-actin as we described previously (44). Extracellular viral DNA was extracted and measured as previously described (41).

### RNA-binding protein immunoprecipitation

RIP was conducted using the Magna RIP kit (Cat #17–700, Millipore) following the manufacturer’s instructions. In brief, Akata (EBV+) cells expressing YTHDF2-Myc or YTHDF2 (K281R/K571R/K572R, RRR)-Myc were treated with IgG cross-linking for 24 h and then lysed using the RIP lysis buffer provided in the kit. A portion of the lysate (10%) was saved as the input sample. The anti-c-Myc magnetic beads (Cat #88842, Thermo Scientific) were washed with RIP buffer and incubated with RNA overnight at 4°C. On the following day, the beads were collected and washed six times with the RIP wash buffer. The enriched RNA-protein complex was digested with proteinase K, and the released RNA was purified using phenol-chloroform extraction. The purified RNA was then subjected to reverse transcription for subsequent qPCR analysis using the primers for *ZTA*, *RTA BALF5*, *BGLF4*, *BLLF1*, and *MALAT1* as we previously described (7, 13)

### mRNA stability assay

Akata (EBV+) cells expressing YTHDF2-Myc or YTHDF2 (K281R/K571R/K572R and RRR)-Myc in six-well plates were treated with IgG cross-linking for 12 or 24 h. The cells where then treated with actinomycin D (5 µg/mL) (Cat #A1410, Sigma-Aldrich) to inhibit transcription. The cells were collected at 0, 2, 4, and 6 h after treatment. The total RNA was extracted with an Isolate II RNA minikit (Bioline) and analyzed by quantitative reverse transcription-PCR with specific primers for *ZTA*, *RTA*, *BALF5*, *BGLF4*, and *BLLF1*. 18s rRNA was used as control (45). All primer sequences are listed in Table S1.

### Structure prediction by AlphaFold-Multimer

AlphaFold-Multimer algorithm (37) was employed to predict protein-protein interactions involving the following sequences: PIAS1 (Uniprot: O75925), YTHDF1 (Uniprot: Q9BYJ9), YTHDF2 (Uniprot: Q9Y5A9), and YTHDF3 (Uniprot: Q7Z739). The detailed procedure was described in the link https://cosmic-cryoem.org/tools/alphafoldmultimer/ (46, 47). Briefly, the amino acid sequences of PIAS1 and YTHDF1, YTHDF2, or YTHDF3 were combined into fasta files, which were then uploaded to the COSMIC2 webserver. This fasta file was used as the input to run the AlphaFold-Multimer tool. Each prediction of protein-protein interaction generated five pdb files. Molecular graphics and analyses of protein interactions were performed with UCSF ChimeraX (48), developed by the Resource for Biocomputing, Visualization, and Informatics at the University of California, San Francisco, with support from National Institutes of Health R01-GM129325 and the Office of Cyber Infrastructure and Computational Biology, National Institute of Allergy and Infectious Diseases. Model 1 of each prediction was used to display PIAS1 interaction with YTHDF1, YTHDF2, and YTHDF3.

### Quantification and statistical analysis

Statistical analyses were performed using a two-tailed Student *t*-test with Microsoft Excel software. A *P* value less than 0.05 was considered statistically significant. The values are presented as means and standard deviations for biological replicate experiments as specified in the figure legends. For RNA decay assay, the figures were created using GraphPad Prism v.9 software. The Fig. 10 was created using BioRender.

## SUPPLEMENTAL MATERIAL

Supplemental material mbio.03168-23-s0001.pdf Fig. S1 to S3; Table S1.

ASM does not own the copyrights to Supplemental Material that may be linked to, or accessed through, an article. The authors have granted ASM a non-exclusive, world-wide license to publish the Supplemental Material files. Please contact the corresponding author directly for reuse.
