# Supplementary material for: SUMOylation of the m6A reader YTHDF2 by PIAS1 promotes viral RNA decay to restrict EBV replication
Source: mBio. 2024 Jan 18;15(2):e03168-23. doi: 10.1128/mbio.03168-23 (PMC10865817; doi:10.1128/mbio.03168-23)
Supplement: Supplemental material — Fig. S1 to S3; Table S1. [file mbio.03168-23-s0001.pdf]

Supplemental Materials

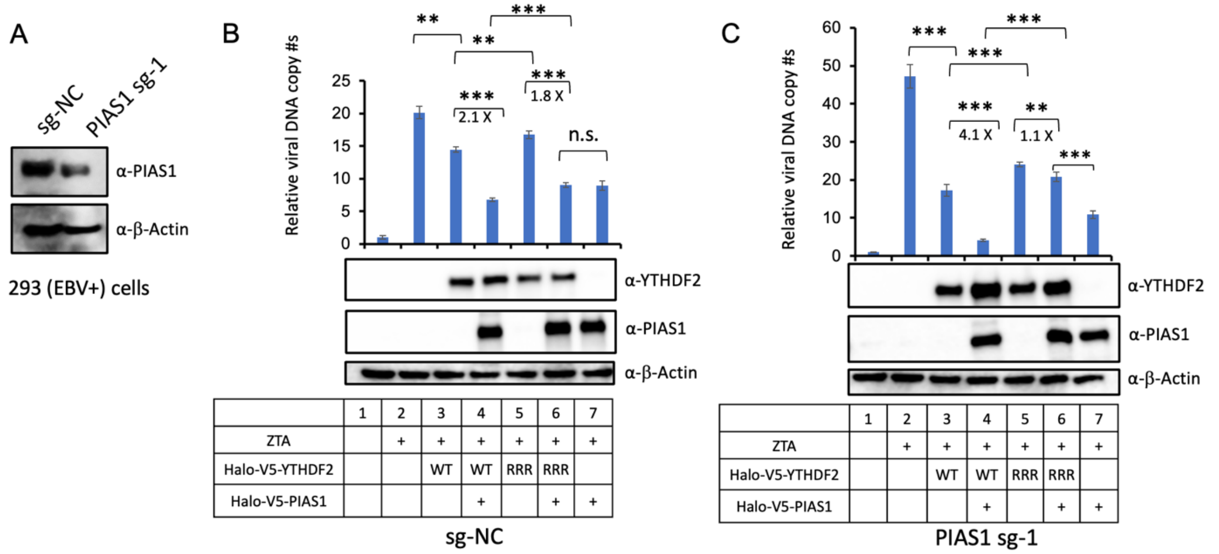

Figure S1. Related to Figure 5.

A. HEK293 (EBV+) cells were transduced with lentiviruses containing CRISPR/Cas9 sg1-PIAS1 (PIAS1 targeting sgRNA) or sg-NC (control sgRNA) to establish cell lines. The expression of PIAS1 was evaluated by WB using anti-PIAS1 antibody. β-actin blot was included as loading control.

B and C. The control (sg-NC) and PIAS1 depleted (PIAS1-sg-1) cells were transfected with ZTA (lytic inducer), PIAS1, and wild-type YTHDF2 (WT) or SUMOylation-deficient YTHDF2 (RRR) as indicated. The relative EBV copy numbers were measured using the qPCR as described in the method. The value of lane 1 was set as 1. The expression levels of YTHDF2 and PIAS1 were monitored by WB. β-actin blot was included as loading control.

Results from three biological replicates are presented. Error bars indicate the standard deviations. $^{**}P < 0.01$, $^{***}P < 0.001$. n.s., not significant.

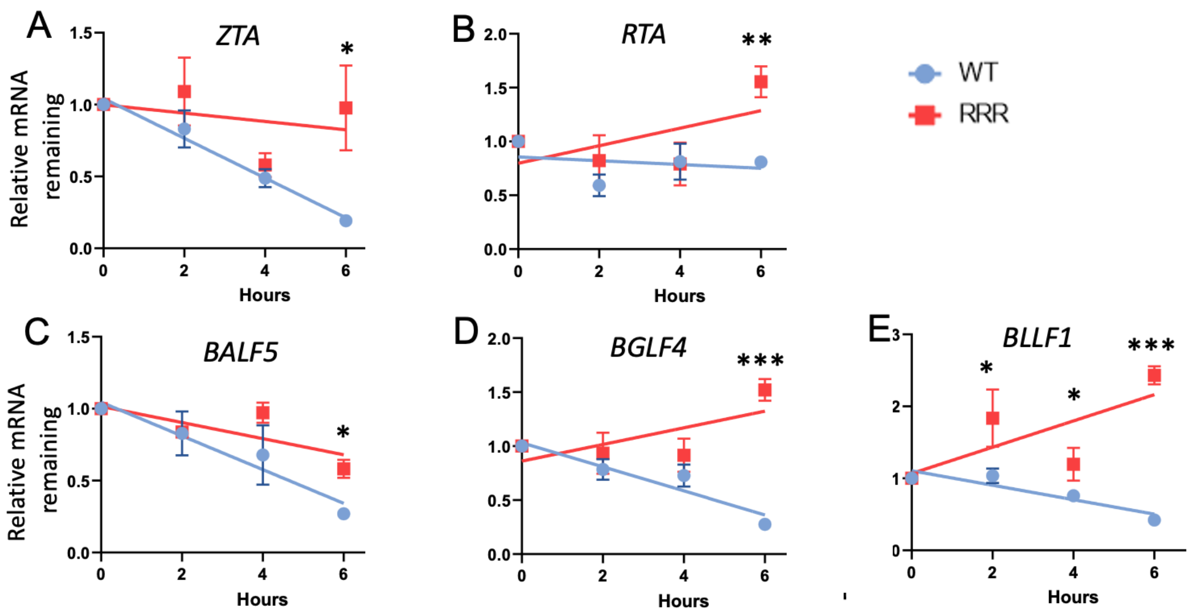

Figure S2. Related to Figure 7

Akata (EBV+) carrying WT YTHDF2 or SUMOylation-deficient mutant (RRR) were lytically induced by IgG cross-linking for 24h. Subsequently, the cells were ttreated with actinomycin D. The Immediate Early (*ZTA* and *RTA*), Early (*BALF5* and *BGLF4*) and Late (*BLLF1*) gene levels were analyzed by qRT-PCR. The relative mRNA level at 0 h after actinomycin D treatment was set as 1. Results from three biological replicates are presented. Error bars indicate Error bars indicate the standard deviations. *$P < 0.05$, **$P < 0.01$; ***$P < 0.001$. n.s., not significant.

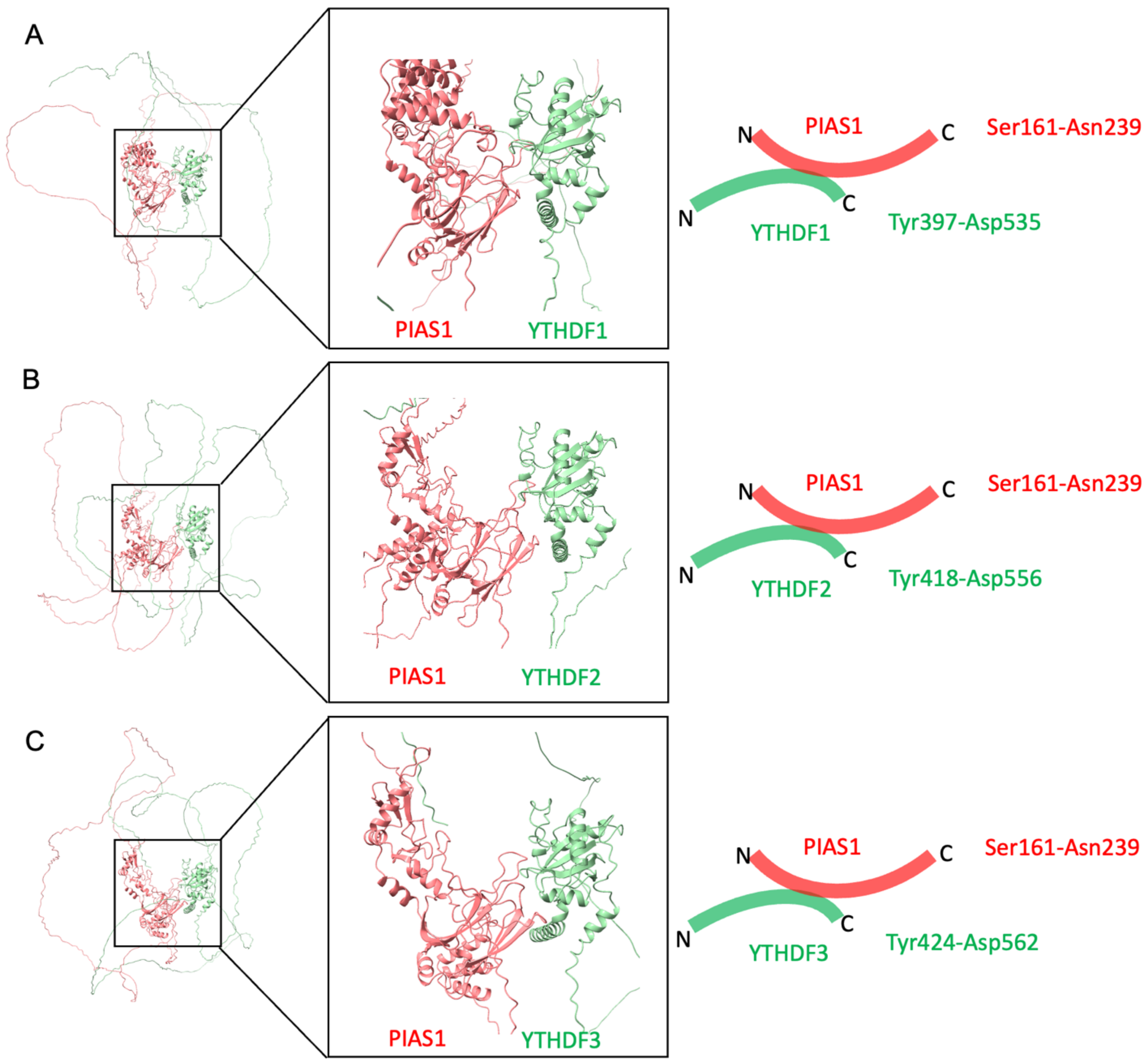

Figure S3. Related to Figures 2 and 8

The AlphaFold-Multimer algorithm was utilized to predict the interaction between PIAS1 and YTHDF1 (A), YTHDF2 (B), and YTHDF3 (C). Among the five models generated, one was selected to visualize the co-structure using the ChimeraX tool. The YTH domains were adjusted

to be in similar positions to illustrate their bindings to PIAS1.The amino acids sequences involved in the protein-protein interactions were illustrated.

Table S1. Primers used in this study.

Primer name	5`-3` sequences
PSF010 (YTHDF2 K571R F)	cttgacgttcctttctaacactttcttcttcctcttggcg
pSF011 (YTHDF2 K571R R)	cgccaagaggaagaagaaagtgttagaaaggaacgtcaag
pSF029 (YTHDF2 K572R R)	gaccttgacgttcccttttaacactttcttcttcctcttggc
pSF030 (YTHDF2 K572R F)	gccaagaggaagaagaaagtgttaaaagggaacgtcaaggtc
pSF031 (YTHDF2 K281R F)	gcaacgggacccctgttatcccaagttccaatatcc
pSF032 (YTHDF2 K281R R)	ggatattggaacttgggataacaggggtcccgttgc
pSF048 (YTHDF2 KK-571/572-RR F)	accttgacgttcccttctaacactttcttcttcctcttggcg
pSF049 (YTHDF2 KK-571/572-RR R)	cgccaagaggaagaagaaagtgttagaagggaacgtcaaggt
pFS_38_F (pHTN-V5-YTHDF1 F insert)	atgggtaagcctatccctaaccctctcctcggtctcgattctacgatgtcggccaccagcgtg gacacc
pFS_38_R (pHTN-V5-YTHDF1 R insert)	ggcccaaatctagatatccgtcattgtttgtttcgactct
pFS_39_F (pHTN-V5-YTHDF1 F vector)	agagtcgaaacaaacaatgacggatatctagatttgggcc
RL0325 (pHTN R vector)	cgtagaatcgagaccgaggagagggttagggataggcttacccatcggttgagctctgaat tcggaagcgat
pFS_41_F (pHTN-V5-YTHDF3 F insert)	atgggtaagcctatccctaaccctctcctcggtctcgattctacgatgtcagccactagcgtg gatcaga
pFS_41_R_N (pHTN-V5-YTHDF3 R insert)	ggcccaaatctagatatccgttattgtttgtttctatttctctccctacgcatggc
pFS_42_F_N (pHTN-V5-YTHDF3 F vector)	gccatgcgtagggagagaaatagaaacaaacaataacggatatctagatttgggcc
pFS_222_F (pLenti-YTHDF3 F)	gatctgccgccgcgatcgccatgtcagccactagcgtggatcaga
pFS_222_R (pLenti-YTHDF3 R)	tcgagcggccgcgtacgcgtttgtttgtttctatttctctccctacgcatggc
pFS_235_F (pLenti-YTHDF1 F)	gatctgccgccgcgatcgccatgtcggccaccagcgtggacacc
pFS_235_R (pLenti-YTHDF1 R)	tcgagcggccgcgtacgcgtttgtttgtttcgactct
pFS_232_F (YTHDF1 K277R F)	cacaggccccctgttatcccaggtgccaat
pFS_232_R (YTHDF1 K277R R)	attggcacctgggataacagggggcctgtg
pFS_233_F (YTHDF1 K551R F)	tctgccgttccctgcgcaccacctcc
pFS_233_R (YTHDF1 K551R R)	ggaggtggtgcgcagggaacggcaga
pFS_234_F (YTHDF3 K282R F)	ttaccactgaccctctttcatcccaagttccaatattcatgt
pFS_234_R (YTHDF3 K282R R)	acatgaatattggaacttgggatgaaagagggtcagtggtaa
pFS_237_F (18s RNA F):	ggttcgattccggagaggg
pFS_237_R (18s RNA R):	tcgggagtgggtaatttgc
